# A Qualitative Evaluation of Clinical Audit in UK Dental Foundation Training

**DOI:** 10.3390/dj5040031

**Published:** 2017-11-10

**Authors:** Peter Thornley, Alyson Quinn

**Affiliations:** 1 Training Programme Director Coventry Foundation Training Scheme, Health Education West Midlands, 213 Hagley Road, Birmingham B16 9RG, UK; 2 Principal Teaching Fellow, Warwick Medical School, University of Warwick, CV4 7AL Coventry, UK; A.Quinn@warwick.ac.uk

**Keywords:** Clinical Audit, Dental Foundation Training, qualitative research, group interviews, continuing professional development, clinical governance, post-graduate dental education, professionalism, management and leadership

## Abstract

Clinical Audit (CA) has been recognized as a useful tool for tool for improving service delivery, clinical governance, and the education and performance of the dental team. This study develops the discussion by investigating its use as an educational tool within UK Dental Foundation Training (DFT). The aim was to investigate the views of Foundation Dentists (FDs) and Training Programme Directors (TPDs) on the CA module in their FD training schemes, to provide insight and recommendations for those supervising and undertaking CA. A literature review was conducted followed by a qualitative research methodology, using group interviews. The interviews were transcribed and thematically analyzed using NVIVO, a Computer-Assisted Qualitative Data Analysis tool. CA was found to be a useful tool for teaching management and professionalism and can bring some improvement to clinical practice, but TPDs have doubts about the long-term effects on service delivery. The role of the Educational Supervisor (ES) is discussed and recommendations are given for those supervising and conducting CA.

## 1. Introduction

Clinical Audit (CA) has been defined as “The process used by health professionals to assess, evaluate, and improve the care of patients in a systematic way in order to enhance their health and quality of life” [1]. It has been recognized as a useful tool for improving service delivery, clinical governance, and the education and performance of the dental team [2,3,4,5]. This paper draws on the results of a qualitative evaluation of young dentists’ experience of audit [5] to provide advice for General Dental Practitioners and others in leadership and educational roles. This should help them use Clinical Audit more effectively. A qualitative methodology was used to ascertain the opinions and thoughts of UK Foundation Dentists (FDs) and their Training Programme Directors (TPDs).

In the UK, Dental Foundation Training (DFT) is a one-year programme following initial qualification that aims “to produce a caring, competent, reflective practitioner able to develop their career in any branch of dentistry to the benefit of patients” [6] and to demonstrate a level of competence appropriate for independent practice. It is mandatory for UK graduates who wish to work in the U.K. National Health Service (NHS) General Dental Service. The Foundation Training Programme includes a placement in a training practice for one year, where the FD carries out clinical work and other duties under the supervision of their Educational Supervisor (ES). There is a day release per week when FDs meet with the other 12 trainees in their locality for a “Scheme” educational study day organized by the TPD. Occasionally throughout the year, all seven training schemes from the region meet and one such day is when an FD with the best audit (judged by the TPD) from each scheme presents their finding to all the FDs (*n =* 90) in the region.

One of the requirements of the curriculum for Foundation Training [7] is that FDs should understand the principles involved in Clinical Audit and be willing to participate in such activities. This should be evidenced by activities such as giving an audit presentation and taking part in study days organized as part of the training programme, as well as observing and taking part in tutorials within their training practice. FDs are required to give an audit presentation with their results at the end of the second term, approximately halfway through the training year. They are responsible for organizing and designing the project, with the assistance and supervision of their ES, who is a senior dentist in their primary care dental practice placement.

The purpose of using CA as an educational tool is to develop the clinical, management, leadership, professionalism, and communication skills which are the major domains of the Foundation Training curriculum. It is a way of reinforcing reflexive practice amongst young clinicians and introducing them to the fact that through their professional careers they will need to monitor their own performance and aim to improve upon it. It emphasizes this essential element of professional practice, whilst providing technical skills through which reflective practice can be implemented.

Considerable time and resources are devoted to Clinical Audit teaching within the foundation training year, but there had not been an in-depth evaluation of the educational benefit of the Clinical Audit teaching or the learning outcomes for Foundation Dentists (FDs).

The purpose of this research is to investigate and evaluate the educational effect of Clinical Audit within Foundation Training in NHS general dental practice. It should be of benefit to anyone involved in training junior clinicians or with responsibility for quality assurance in healthcare service delivery.

The study was conducted using a Qualitative Methodology, appropriate for assessing complex educational systems. The experiences of the Foundation Dentists themselves were the focus of the study.

Although qualitative research is widely used in sociological and educational research, it is used less often in medical and dental disciplines, where a quantitative approach is more frequently utilized. Indeed, some policymakers and clinicians have expressed doubt that qualitative research can provide anything useful [8]. However, for complex social interactions, such as educational processes, a qualitative approach is more appropriate, and the rationale for its use is provided in the Methods section.

### Clinical Audit

**Figure 1:**
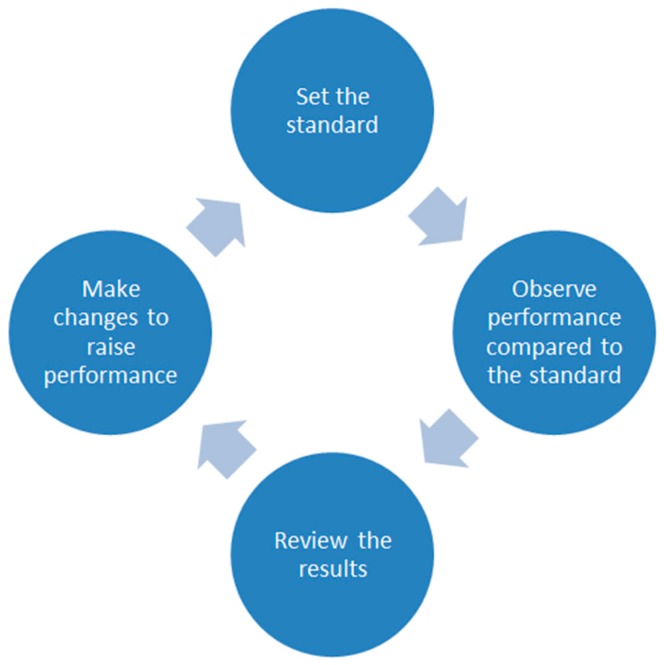
The audit cycle.

The principles of Clinical Audit are to set a clinical standard (preferably with reference to an external guideline), collect data and review existing practice with reference to that standard, act to improve practice, and then re-audit [9] (Figure 1). Several steps are involved, and one purpose of this research was to find out how FDs worked through the process. Which areas did they find difficult and how could tutors and trainers facilitate their progress and educational development?

Subjects for investigation were:

- The choice of topic and getting started. In Foundation Training, the FDs can study an audit topic of their choice. However, the subject must fall within the Department of Health’s definition of CA as “… the systematic, critical analysis of the quality of dental care, including procedures and processes used for diagnosis, intervention and treatment, the use of resources and the resulting outcome and quality of life as assessed by both professionals and the patient…” [10]. This definition includes broader topics such as practice administration, procedures for dental radiography, record keeping, antibiotic prescribing, and waste disposal. These and many other topics have been audited by dentists and Foundation Dentists. Are some subjects more useful than others? Are some topics more difficult to execute?
- Setting a standard. Ideally, this should involve reference to an external clinical guideline for good practice. An element of research is required here. However, sometimes such guidelines may not exist or may not be accessible to the auditor. Furthermore, in his review of Clinical Audit, Fleming [2] noted that the initial guidance sent out to dentists by the UK Department of Health was not clear about setting standards for CA. Operationally, standards have been reduced to percentage “targets” which can be difficult to understand and apply at the outset of an audit, especially if the first cycle is a baseline study. A better approach is to use grading criteria, which are available for some clinical procedures e.g., in the Faculty of General Dental Practice booklet “*Standards in Dentistry*” [11]. Team meetings can be used to refine and apply these standards to the local practice, and after the first cycle a percentage target can be identified, related to the performance recorded. Does this confusion still exist? How do FDs set a standard and what could be done to clarify and facilitate the process?
- Data collection. The next stage in the audit cycle involves collecting data to compare the procedures in the practice with the standard set. Having decided upon the specific items to be recorded, the auditor must design or use a pre-existing data collection sheet. This may require Information Technology (IT) and other skills. Collecting the data itself requires discipline and time management skills. Does audit develop teamwork and management skills for young dentists?
- Making changes. Having identified areas of good and inferior performance, the auditor should communicate this to the dental team, via staff or one-to-one meetings, possibly supported with written or projected material. For many FDs, this is the first time they have led a team meeting, and thus their feedback on their fears and barriers to success as well as the support they would like will be interesting to their supervisors and others who may be conducting audit themselves.
- Re-audit. Having implemented changes, the final stage is to repeat the cycle to see if performance has improved.

The purpose of this paper is to provide insight and recommendations for those supervising others or conducting audit themselves. The overall aim is to improve service delivery and educational progression by reflecting and acting on the evaluation. A secondary aim is to demonstrate the use of qualitative research methodology which will be described in more detail in the Methods section. The qualitative nature of the study allows investigation into how FDs deal with problems and difficulties that arise in the process of audit. The potential effects of audit on the dental team are investigated, and insight is provided into the support required for those conducting audit. Finally, suggestions are made for building on the benefits of Clinical Audit.

## 2. Methods

### 2.1. Qualitative Research Design

A qualitative research design was used for this study because it provides a better understanding of complex social interactions than quantitative designs [8]. The disadvantage of this methodology is that it can be subjective, but it allows a focus on meaning—in this case, concerning what FDs think about clinical audit. A naturalistic [8] approach was used to study Foundation Training Groups in their usual social context, and the design was flexible so that it could be modified as the study progressed, responding to the focus group interviews.

The theoretical approach used was one of “critical realism” [12], assuming that aspects of foundation training can be identified and understood, to some degree, but not assuming that the way the world of FD training works will be exactly the same at different times and places. The data was examined for patterns and two FD groups and a group of TPDs were interviewed to provide some triangulation.

### 2.2. Methods

A literature review was conducted and then focus group interviews [13] were conducted. Purposive sampling of pre-existing FD groups from a UK regional FD scheme was made. The aim of purposive sampling is to focus on the characteristics which may affect perceptions. The groups were chosen to reflect the diversity of ethnicities in the region [5]. The interviews [14] were carried out and digitally recorded. The interviews were transcribed verbatim and analyzed using a “Framework Approach” [15] within NVIVO Computer-Assisted Qualitative Analysis Software [16]. Ethical guidance was received from a supervising university and from the local NHS research and development lead.

Provisional prompts for the interviews were developed, based on the work of Donabedian [17] and Cannell [18]. The question prompts were:

- What was the name and place of your undergraduate training? Did you undertake CA as an undergraduate?
- Tell me about the structure of your CA teaching as an FD. Was it easy to engage/understand?
- Process of audit—can you tell me about the following?
  ○ What were the teaching principles?
  ○ Designing your audit.
  ○ Data collection and analysis.
  ○ Report generation and giving feedback to your team.
  ○ Presentations.
- What was the impact and change to your practice?
- How useful was it overall?
- Do you have any advice for improvement or modification?

### 2.3. Data Analysis

The interviews were transcribed within NVIVO (QSR International (UK) Ltd. Daresbury, Cheshire, UK) and then the data was coded, categorized, and indexed. A thematic analysis was undertaken, and a framework was developed. Relationships and comparisons were drawn out within and between the interview data. Theory was developed to answer the research questions using the principles described by Gibbs [19].

## 3. Results

### 3.1. Choosing a Topic and Getting Started

Embarking on CA provided some challenges for FDs. They wanted to choose a topic that was original and important to them and for the practice where they were working. “*It has to be your own project … you have to learn by doing it. If someone just spoon feeds you, here's the research, here's the first cycle, here's the data collection… you’re not going to learn*” FD.

TPDs recognized that the topic had to be manageable and easy to execute. “*Well that sounds like a fantastic idea, but have you thought about how detailed your data capture sheet is going to be…*.” TPD.

These three criteria of originality, importance, and manageability could be mutually exclusive. An important topic is likely to have been covered before, or an original and interesting audit may require data collection or resources that are beyond the FD at this stage. A key role of the Educational Supervisor (ES) is to manage the expectations of the FD about their project. TPDs pointed out that it can be useful to revisit a subject that has been audited before, to see if improvements have been maintained. The topic chosen must have sufficient data available to allow its investigation. For instance, an audit investigating the marginal fit of indirect restorations could fail because FDs carry out relatively few such restorations in the early months of their training, unless they can involve other clinicians in the practice to provide a large enough data set. FDs considered “*off the shelf*” audits “*boring*” and not always relevant to their practice situation. TPDs recognized the value and skills developed by designing and executing an audit as opposed to simply collecting data for a pre-existing CA.

One TPD identified two types of audit: Continuous Rolling Audits—conducted to maintain standards, and Diagnostic Audits—performed to identify a problem within the practice. In her opinion, diagnostic audits provide a richer learning experience and their importance was also recognized by FDs for practice and personal development: *“I saw a lot of caries in young children, carious 6’s and simple things… Couldn’t they have benefitted from fissure sealants or dietary advice?”* FD.

*“Well, one of the issues in my practice was, erm, I wasn’t printing FP17 DC* (Treatment planning) *forms—I thought I was but then when I was going back I was like, “Oh my God, I haven’t done that.”*.

FD

FDs and TPD reported a wide range of topics that had led to successful audits. These included:

- Items from the guideline “*Delivering better oral health*” [20] such as the application of fluoride varnish, patient advice, etc.
- Antibiotic prescribing
- Infection control and compliance with the *HTM 01 05* [21] guideline
- Clinical waste disposal
- Record keeping
- Recall intervals
- Caries diagnosis
- Periodontal Treatment

### 3.2. Time and Self-Management

FDs think that CA helps them develop their time and self-management skills: “*It was quite nice that we were just left to choose what we wanted to do… it just helps us with our time management*”*.* FD. As discussed above, trainers have a role in balancing the complexity and manageability of audit. If FDs get this balance wrong, it can be stressful, and they can be tempted to fabricate data: “*You just think, oh my God, people aren’t handing out that, people aren’t doing this, it’s got to be in for this time, why don’t I just make it all up? Not that I did, but…"* FD. Better strategies for time management included delegation and involving other members of the practice to help with data collection. The ES has a role in ensuring that some time is set aside for conducting the audit, but there is also a place for developing professionalism and realizing that some administrative and research tasks may need to be conducted in one’s own time. There were different attitudes to using clinical time to conduct audit. FDs are in salaried positions and their salaries are not dependent on their clinical output. Their attitude to the use of clinical time reflects this: “*I mean, just book off some clinical time just to do it. It doesn’t really affect anyone!*” FD. Not all FDs could carry out audit in their clinical sessions and had to conduct the work either in their own time or when a patient had cancelled. ESs should be aware of differing expectations and clearly lay out what the practice policy is regarding the use of surgery time to conduct the audits. Some FDs put a lot of their own time into the project, staying behind after work “*…with the cleaners*” but they felt they had produced a worthwhile result: “*I spent a lot of time … it was quite exhausting, but in the end, when I did my cycle 2 it was in a lot more orderly fashion and it made it so much easier for nurses to collect and screen for out of date instruments.*” FD.

Others put less effort in and realized that their audits were not as valuable: “*Just kind of questionnaires, I’d just kind of collect them up and process them, so it wasn’t very time consuming*”*.* FD. *“I know other people’s audits made a difference, but mine, not much, no…”* same FD.

FDs appreciate the support of their ES in conducting the audit and helping to involve the team, but they do not want their trainer to take over: “*It was my audit, it was my idea and I was really, like, kind of, pushed for myself to do it. And I kind of, got him (ES) on board, but as soon as I had chosen, he was like, “Right and now let’s do this.” And I felt like, whoa, let’s tone it down a little bit.*” FD.

### 3.3. Support and Resources

A wide range of resources were used by FDs to produce their audit. These included guidance and guidelines from bodies such as the British Dental Association and the Faculty of General Dental Practitioners. Print and library resources were used, together with print-outs from their trainers or TPDs. Electronic search tools such as Google scholar and PubMed were used, but FDs had problems accessing papers and the library subscriptions they had as students were no longer available. Two TPDs put on a library training course which included information on how to access NHS Athens accounts, etc., but a general complaint from FDs was that they found it difficult to access original papers. “*I don’t have access to Medline or like Ovid SP anymore, so… trying to find ones myself, and abstracts, and ones that weren’t texts and stuff… it’s difficult. Not as straightforward as it used to be. In dental school it was really easy, you had access to everything but, yeah, it’s more difficult.*” FD. They expressed some distrust of clinical guidelines, reflecting the mistrust of published guidelines discussed by Bilawka [22] and Ferlie [23]. “*I think guidelines are available, but research is hard* (to get).” FD.

The most valuable resources for many FDs were their dental team, colleagues, and ES. Those that harnessed the support of colleagues produced more productive audits and felt that the project was more worthwhile: “*Once the practice principal was on board, I raised it at a staff meeting and everybody was like, “Yeah let’s get together and do this”. So that was quite positive*” FD. “*I found the team pretty supportive, and they want to know the results as well*.” FD.

### 3.4. Standards and Targets

Fleming’s observation [2] that there was confusion between setting a standard for audit and providing a target was replicated in this study—for both FDs and TPDs. Fleming states that a standard should be external and align with evidence-based good practice. It should include explicit criteria e.g., “No overlap on Bitewing Radiographs” or “Patients in pain are seen within 24 h”. These quality criteria should be defined at the outset of audit; however, it is not necessary to produce arbitrary percentages of targeted achievement. At the end of the first cycle, it will be possible to see in what percentage of cases the standard was met, and in subsequent cycles this could be used as a target for improvement. Adopting Fleming’s advice would reduce the confusion for FDs, ESs, and TPDs. “*I think this 80% figure tends to crop up quite a lot because they can’t sit around say 60% or 70, 90 to 100 sounds too “gold standard” and erm, er, 80 seems to be a safe number.*” TPD.

### 3.5. Collecting and Analyzing Data

As noted above, ESs should encourage FDs to involve the whole team, including nurses, associates, and others in their data collection. FDs found that their projects worked well when they were incorporated with other initiatives such as “Smile month” that were relevant to the Practice team. TPDs did not think that complex statistics were required for analyzing data, although a large enough sample size was important. FDs usually had the IT and presentation skills required to analyze and present data—TPDs thought the FDs’ skills in this area were better than their own. FDs recruited assistance from several sources, including family members, friends, and colleagues, to help with data analysis. They did not want more lectures on the topic, but would have found published notes or aide-memoires helpful when it came to things like designing and using spreadsheets.

### 3.6. Presentations and Feedback of Results

For audit to be useful, the results need to be fed back to the team and recommendations should be made for improvement. Usually this will involve leading a team meeting, which is a requirement in the FD schemes investigated. One-to-one discussion with a team member may be required. FDs find these team meetings challenging and value the support of their ES. They find personal discussions with team members, especially if there is an area of underperformance to discuss, even more difficult. TPDs recommended that FDs anonymize their data to avoid being judgmental about individuals in a team meeting. Some FDs were also aware of the need to avoid *“naming and shaming”* poor performers in a public forum. Sometimes the poor performer was the ES, but usually the trainers had a mature attitude to objective criticism. TPDs thought that some training on leading a team meeting would be useful for FDs, and some trainers provided this. Support when having *“awkward conversations”* with team members was valued by FDs*.* “*My trainer recognized that some of the conversations would be awkward with some of the staff members, so he kind of selected like, I’ll sit in with you on this conversation that you are going to have with this dentist, and we’ll talk to him together about it. So, I think that’s quite useful*”*.* FD.

After the audit is complete, the FDs are required to present a summary to the other FDs in their scheme. TPDs were aware that this might raise concerns for trainers: “*Erm. I have a feeling there was areas they (trainers) didn’t want highlighting as far as deficiencies were concerned—erm they didn’t want their practices to look bad*” TPD. However, TPDs considered that audits that exposed a problem and dealt with it demonstrated good practice: *“In one year the regional winner was an audit that did expose some, … you know, deficiencies in the practice, but actually they were applauded for the trainer being prepared to let that go public, and the fact that it was very definitely an audit that showed improvement in clinical care.*” TPD. Programme Directors were aware that some subjects may be considered “*out of bounds*” by trainers*—*an example was given of an FD “*warned off*” conducting an audit on the treatment of periodontal disease because the practice did not have the resources to comply with a national guideline.

## 4. Discussion

Qualitative data contains circular linkages between the methods, data collection, and analysis, so some discussion of the findings has already taken place within the introduction and results section [24].

FDs report an improvement in both their educational knowledge and clinical behavior by conducting their own audit and listening to other FDs’ presentations. Audit can be a way of translating new knowledge or guidelines into general practice. Whether this change will be sustained is doubted by some of the TPDs interviewed, and is not answered by this study—as such, it remains an area for further research.

FDs often wanted to do something *“original”* for their audit, but TPDs were concerned that their audits are discrete cycles. Some TPDs think that FDs should be encouraged to go back and repeat previous audits to ensure a spiral of improvement. FDs would not be enthusiastic about this idea as they see repeating “*audits that have been done a hundred times before*” as boring. One TPD considers *“diagnostic audits”* a much better educational experience than carrying out *“continuous rolling audits”.* This should be borne in mind when prescribing compulsory audit to dental teams—it may be more beneficial to allow some leeway in the design and focus of a study rather than prescriptively requiring data collection for a pre-existing study.

Many FDs chose managerial or administrative topics, such as record keeping, instead of a clinical skill, to audit. This was reinforced by TPDs who saw clinical topics as being complicated and anticipated difficulty in gathering enough data. FDs had trouble in finding an external standard for some clinical topics. “*Some of them will say, “Shall I not do this now because I can’t find anything on it?” And I'll say,* “*That’s not a reason not to conduct the audit. If someone else hasn’t done it or there’s no National guidance on it, there’s still a valid reason … Think of why you’ve chosen this topic.*” TPD. There are clinical guidelines available which set out standards and criteria for clinical procedures. An example is *Standards in Dentistry* [11], published by the Faculty of General Dental Practice. Being aware that a proportional target does not have to be produced until after the first cycle may help FDs with their audit design [2]. Conducting collaborative audits with other clinicians in the practice or with other FDs may provide larger datasets, allowing the auditing of clinical topics. These steps could simplify the process of auditing clinical topics that might be more beneficial, relevant, and interesting for FDs than the TPDs’ focus on managerial topics.

This is not to say that the effective management of complex record and administration systems is unimportant. As these do not tend to be the focus of undergraduate training, it is highly appropriate for junior clinicians to have the opportunity to learn such skills in a supervised environment.

One of the objectives of this study was to find out how FDs respond to differences in clinical opinion. The results show that many react in a mature and thoughtful way. Variance in clinical behaviour and knowledge often initiated an FD’s audit. They researched the literature and sought guidelines on what was considered good practice. They audited their own and their colleagues’ behaviour and then presented their findings, usually in a non-judgmental way, at staff meetings. They had some difficulty accessing academic databases and research literature—it would be worth following the lead of the TPD who introduced a session on library skills and electronic resources in the other teaching groups.

Johnston et al. [25] noted several barriers to successful audit, and some of these were also identified in this study:

- Problems between groups and group members—FDs were not always sure that other team members were completely engaged and resorted to bribes such as snacks to encourage participation. Some practice teams evidence *“audit fatigue”.* “*Say you’ve been an associate or a practice owner for 10 or 15 years, you've done the same thing for 15 cycles, 15 years in a row… you can feel it on the weight of the papers…*” FD.
- Difficulties with overall planning and organizational impediments.
- Lack of expertise in project design (compare with the difficulty FDs had with topic selection and getting started).

Many of the FDs interviewed dealt with these difficulties in a professional manner, and they could provide the “Aha” moment when an intellectual puzzle was identified [8]. For the more engaged students, this in turn led them on to thinking and designing strategies to overcome the problem (e.g., leading them to discuss with their trainer how to speak to a non-compliant team member.)

The interaction with trainers was interesting, with FDs wanting help when they needed it, but not wanting their trainers to lead the study or take over the data analysis. There is evidence that FDs’ learning, understanding, and meaning grow out of their social encounters in accordance with Vygotsky’s [26] theory of social constructivism. The FDs learn in a “Zone of Proximal Development”, which is “*… the distance between the actual developmental level as determined by independent problem solving and the level of potential development as determined through problem solving under adult guidance, or in collaboration with more capable peers*”*.*

There is a lesson for trainers and TPDs as well, in that FDs want support and advice when they need it, but not too much. “*I don’t want them to be like, trying to spoon feed us… There's got to be a bit of, you know, you’ve got to do this for yourself.*” FD. FDs are finding their feet as independent professionals and they want some space to develop their own ideas. This fits with Wood’s idea of “scaffolding” [27]—the idea that a teacher or more competent peer gives help to the student in his or her Zone of Proximal Development when needed and then gradually reduces the help as the student becomes more competent.

## 5. Conclusions

### 5.1. Benefits of Clinical Audit

Several benefits of Clinical Audit were identified. It encourages FDs to communicate with patients and colleagues and can help them learn to address challenging situations such as *“awkward conversations”* about sub-optimal performance. Examining associations between administrative data, patient records, and a clinician’s own practice reveals much about the complex nature of patient care within both a fee-for service or subsidized healthcare system. Clinicians should consider such issues especially during such a formative period of their careers.

CA can encourage teamwork and provides an opportunity to lead team meetings and develop managerial and team training skills. It can encourage association with professional bodies and an improvement in clinical practice for the individual dentist and the team: *“Some of the audits have been really novel ideas… get members of the team on board, then it’s not such a one-person task. You know, asking everyone to do something, it’s more like a team than a practice”* FD.

### 5.2. Overcoming Barriers and Problems

The choice of topic needs to be considered carefully, and the supervisor should ensure that it is not too complex or difficult to collect data. Interesting and novel topics motivate FDs and provide a rich learning experience, but they need guidance to prevent them becoming too complex and unmanageable. Working within a tight timeframe can be stressful for FDs and can tempt them to behave unprofessionally. Encouraging teamwork, delegation, and providing some protected time to conduct the audit can mitigate against this. FDs require access to evidence-based guidelines and research papers, and some training on how they can access this material when they no longer have the support of a university library may be necessary. In the UK, the NHS provides some Athens access, and open source journals are becoming more available. Trainers and Educational Supervisors should not “*Sweep things under the carpet*” if areas of sub-optimal performance are identified, because Programme Directors consider it good practice to identify and improve poor practice. In their own words, FDs find clinical audit beneficial: “*By taking this audit on board ourselves and having carried it out, we’ve had to work with it, in our practices, and as a team to do it. So, it does give you some confidence to go into a job and say, ’Look, I’ve done this, I achieved it.*” FD.

### 5.3. Application of Findings and Areas for Further Research

This study has highlighted some of the sensitivities and pragmatic challenges of monitoring clinician performance and the safety and quality of health services. Not only are there sensitivities comparing results between practitioners, but these can have profound effects in terms of a practice’s business model. Using Clinical Audit can provide insight for managers into the expectations of patients. Asking junior clinicians to identify, discuss, and remediate instances of sub-optimal performance is daunting for them, but can provide a very positive educational experience for the whole practice, if managed sensitively and supported by senior members of the team.

There is evidence from this study that Foundation Dentists learn in a “Zone of Proximal Development” in accordance with Vygotsky’s [26] theory. Trainers and Programme Directors should bear this in mind when teaching and developing the educational programme.

Foundation Dentists gained a great deal of benefit from seeing an example of an audit presented by FDs from the previous year’s group. Many of them did not understand the concept and process of audit until they had seen such an example. It would be worthwhile re-arranging the audit presentations so that winners present to the following year’s Foundation Dentists, or else involving an FD from the previous year on the introduction day.

The associated principle of “scaffolding” [27] should also be borne in mind. Teaching works best if help is given to move FDs slightly further on from their existing zone of knowledge, gradually withdrawing help as they become more competent. Too much intervention can be as off putting to FDs as too little. FDs are developing their skills as independent practitioners, and want support from their trainers, but do not want *“spoon-feeding”.* Trainers should work in the “Goldilocks Zone” when providing support: not too much, nor too little, but just the right amount of help when needed.

How trainers gauge that they are working in the “Goldilocks Zone” would be a fruitful area for future research. Factors such as the trainee’s previous experience and their learning style could be important. The listening skills of the trainer and the development, application, and evaluation of their mentoring abilities are other topics that warrant further research.
